# The Effects of Autophagy and PI3K/AKT/m-TOR Signaling Pathway on the Cell-Cycle Arrest of Rats Primary Sertoli Cells Induced by Zearalenone

**DOI:** 10.3390/toxins10100398

**Published:** 2018-09-28

**Authors:** Bing-jie Wang, Wang-long Zheng, Nan-nan Feng, Tao Wang, Hui Zou, Jian-hong Gu, Yan Yuan, Xue-zhong Liu, Zong-ping Liu, Jian-chun Bian

**Affiliations:** 1 College of Veterinary Medicine, Yangzhou University, Yangzhou 225009, China; wangbingjieyzh@outlook.com (B.-j.W.); wanglongzheng@vet.k-state.edu (W.-l.Z.); fengnannan8@outlook.com (N.-n.F.); wtao6550@yzu.edu.cn (T.W.); zouhui@yzu.edu.cn (H.Z.); jhgu@yzu.edu.cn (J.-h.G.); yuanyan@yzu.edu.cn (Y.Y.); Liuxuezhong68@yzu.edu.cn (X.-z.L.); liuzongping@yzu.edu.cn (Z.-p.L.); 2 Jiangsu Co-Innovation Center for Prevention and Control of Important Animal Infectious Diseases and Zoonoses, Yangzhou 225009, China; 3 Joint International Research Laboratory of Agriculture and Agri-Product Safety, the Ministry of Education of China, Yangzhou University, Yangzhou 225009, China

**Keywords:** Zearalenone (ZEA), Sertoli cells (SCs), cell cycle, G2/M arrest, Autophagy, PI3K/Akt/m TOR signaling

## Abstract

A high concentration of Zearalenone (ZEA) will perturb the differentiation of germ cells, and induce a death of germ cells, but the toxic mechanism and molecular mechanism remain unclear. The Sertoli cells (SCs) play an irreplaceable role in spermatogenesis. In order to explore the potential mechanism of ZEA male reproductive toxicity, we studied the effects of ZEA on cell proliferation, cell-cycle distribution, cell-cycle-related proteins and autophagy-related pathway the PI3K/Akt/mTOR signaling in primary cultured rats SCs, and the effects of autophagy and PI3K/AKT/m TOR signaling pathway on the SCs cell-cycle arrest induced by ZEA treated with the autophagy promoter RAPA, autophagy inhibitor CQ, and the PI3K inhibitor LY294002, respectively. The data revealed that ZEA could inhibit the proliferation of SCs by arresting the cell cycle in the G2/M phase and trigger the autophagy via inhibiting the PI3K/Akt/m TOR signaling pathway. Promoting or inhibiting the level of autophagy could either augment or reverse the arrest of cell cycle. And it was regulated by PI3K/Akt/m TOR signaling pathway. Taken together, this study provides evidence that autophagy and PI3K/Akt/m TOR signaling pathway are involved in regulating rats primary SCs cell-cycle arrest due to ZEA in vitro. To some extent, ZEA-induced autophagy plays a protective role in this process.

## 1. Introduction

Zearalenone (ZEA), which has always been called F-2 toxin, is synthesized by the polyketide pathway, a serious mycotoxin existed in human food and animal feed [1,2]. Many studies have shown that ZEA and its dangerous secondary fungal metabolite can induce reproductive toxicity, immunotoxicity, DNA damage, and carcinogenicity qualities [3,4]. Studies reported that female animals are more sensitive to ZEA compared to male animals, so studies of ZEA have mainly concentrated on its toxicity in the female endocrine system [5,6]. ZEA can lead to female reproductive disorders such as abortion, stillbirth, castration, and sow false heat, or even potential danger to public and environmental health [7]. Studies also have shown that ZEA has estrogenic activity that acts on the reproductive system of animals by binding to estrogen receptors and results in a variety of estrogenic effects [8,9]. In recent years, its estrogen-like effects of reproductive toxicity in male animals has attracted more attention [10]. Qing [11] showed that ZEA can significantly reduce testosterone secretion in Leydig cells by the cAMP pathway in both luteinizing hormone and human chorionic gonadotropin environments. The primary rat Leydig cells treated with ZEA result in viability inhibition. The result also revealed that low or high doses of ZEA can activate autophagy and apoptosis in Leydig cells and autophagy can delay the occurrence of apoptosis in Leydig cells. Therefore, autophagy has an important protective effect against cytotoxicity in a certain extent [12].

SCs comprise a good model for evaluating toxicity in the male reproductive system in vitro and vivo, and revealing the mechanism of ZEA in male reproductive toxicity. For male animals, the number of SCs is a key determinant about the size of the testis and spermatogenesis in adulthood [13,14]. Studies have shown that SCs are the most important target cells of the male reproductive system for many environmental pollutants, and germ cells around of SCs are considered a barrier that protects spermatogenesis from harmful influences [15,16]. In our preliminary studies [17,18], we chose TM4 cells (mouse Sertoli cell line) as a model, as a result, damage to the cytoskeletal structure and disturbance of specific secretory functions of TM4 cells were found, which may be an underlying cause of ZEA-induced reproductive toxicity in the female endocrine system. The result indicates that a high dose of ZEA also can perturb the differentiation of germ cells and induce a death of germ cells in an extent, but the molecular mechanism remains unclear.

Cell cycle, the basic process of cell life activities, is under the control of endogenous and exogenous factors in a certain order to complete the cell division and reproduction process. Many researchers have found that autophagy play an important role in cell cycle and apoptosis regulation [19,20]. It plays a role in maintaining a defensive and stress-regulating mechanism of cellular homeostasis in the body [21,22]. The Autophagy related pathways, that PI3K/Akt/m TOR signaling pathway mediated cell proliferation, differentiation, migration, apoptosis, autophagy, and other physiological functions targeted at m TOR [23,24].

Our previous study [18] has shown that ZEA can interfere the differentiation of cells in high concentration, and induce a death of germ cells to a certain extent. However, the underlying molecular mechanism of the cytotoxicity is unclear. In this study, we used primary SCs of male Wistar rats to elucidate the effect of autophagy and PI3K/Akt/m TOR signaling pathway on the cell-cycle arrest induced by ZEA, and provide a theoretical basis for the prevention and treatment of ZEA poisoning.

## 2. Results

### 2.1. Cytotoxic Effect of ZEA in SCs

**Figure 1:**
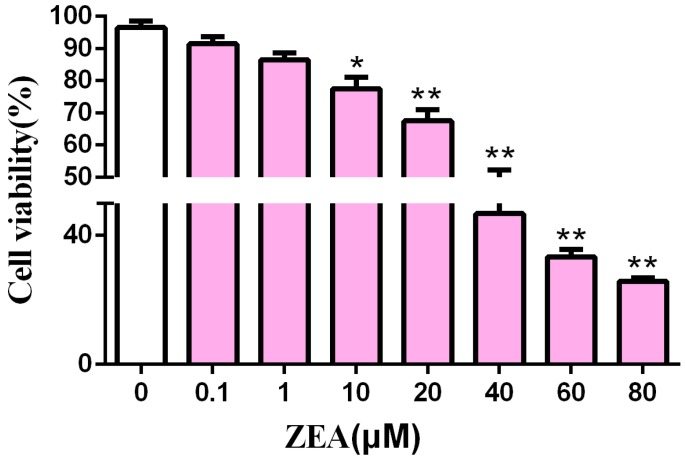
Effects of Zearalenone (ZEA) treated on Sertoli cells (SCs) viability. * *p* < 0.05, ** *p* < 0.01 versus control.

SCs were treated with different concentrations of ZEA. CCK-8 was used to determine the effects of different concentrations of ZEA on SCs activity (Figure 1) after a 24 h experiment. As the ZEA concentration increased, the survival rate of the SCs decreased, this shows a dose-dependent relationship. At 20 μM, cell viability was significantly decreased (*p* < 0.01); at 40 μM, the half-maximal inhibitory concentration was reached. In the following test, 10 μM and 20 μM ZEA were selected as the concentrations.

### 2.2. The Effects of ZEA on the Cell Cycle Distribution and the Cell Cycle Associated Proteins in SCs

When SCs grew in the phase of logarithmic, they were treated with different concentrations of ZEA for 24 h. The changes of the cell cycle were detectable on flow cytometry (Figure 1B). As the concentration of ZEA increased, the percentage of SCs in the G0/G1 phase decreased, while the percentage in the G2/M phase increased. When the ZEA concentration was 10 μM, the ratio of S phase change was not obvious, whereas the proportion of cells in the G2/M phase increased significantly (* *p* < 0.05); when the concentration of ZEA was 20 μM or 30 μM, the G0/G1 ratio decreased significantly (* *p* < 0.05), while that of the G2/M phase increased dramatically (** *p* < 0.01). These results indicate that ZEA could induce SCs cycle arrest in the G2/M phase and inhibit SCs proliferation in a dose-dependent manner.

**Figure 2:**
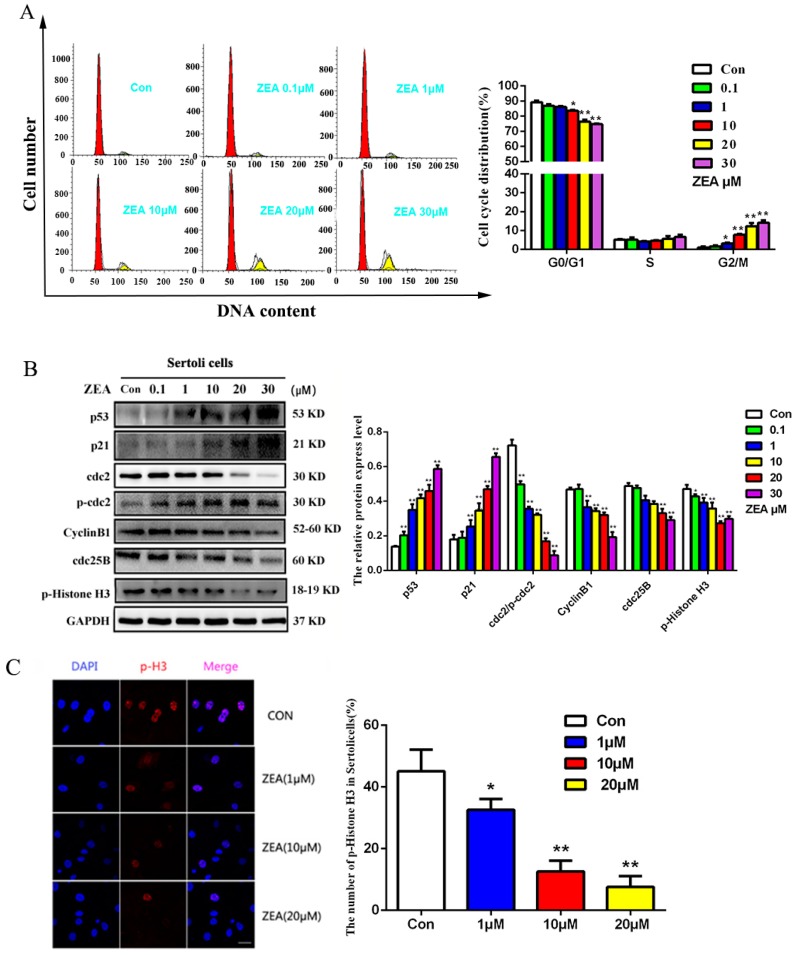
ZEA induced G2 phase arrest in SCs. (**A**) After treatment with different concentrations of ZEA for 24 h, the cell cycle changes were detectable by flow cytometry; (**B**) The influence of protein expression of SCs in the G2/M phase versus control (* *p* < 0.05, ** *p* < 0.01); (**C**) Immunofluorescence analysis of the number of sensitive mitotic cell marker p-Histone H3–positive cells after ZEA treatment.

To elucidate the possible mechanisms which contribute to the induction of G2/M phase arrest by ZEA in SCs, we analyzed the expression levels of cell cycle–associated regulatory proteins for G2/M transition (cdc2, cdc25B, and Cyclin B1) by Western blotting (Figure 2B). Cdc2, Cyclin B1, and cdc25B proteins, which playing important roles in G2/M cell cycle progression, were significantly decreased by ZEA in a dose-dependent manner. ZEA also increased the protein expression levels of p53 and p21 (Figure 2B); compared with the control group, p-Histone H3 expression was significantly decreased, which confirmed that ZEA could markedly decrease the proportion of M phase cells and significantly inhibit SCs proliferation in a certain concentration range.

P-Histone H3 (Ser-10) is a marker protein of the mitotic phase of cells. The positive expression of fluorescein isothiocyanate (FITC)-labeled p-Histone H3 was fluorescent red on confocal microscopy located in the nucleus of SC cells in the division phase, while the nucleus labeled with DIPA showed red fluorescence. The immunofluorescence results showed that after 20 µM ZEA treatment, the number of cells expressing p-H3 decreased significantly compared with that in the solvent control group, further confirming that the number of cells in the M phase could be reduced by ZEA treatment (Figure 2C). The results of flow cytometry (FCM), Western blotting, and immunofluorescence (Figure 2A–C) showed that ZEA treatment could induce G2/M arrest in SCs.

### 2.3. ZEA Could Trigger the Autophagy in SCs

**Figure 3:**
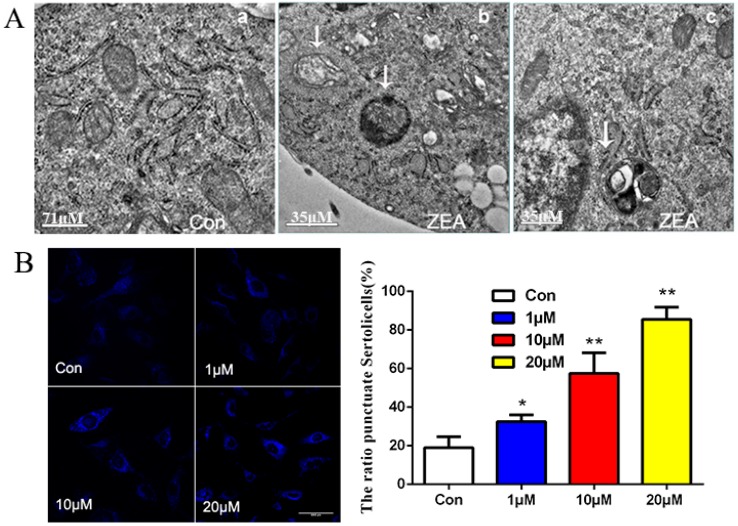

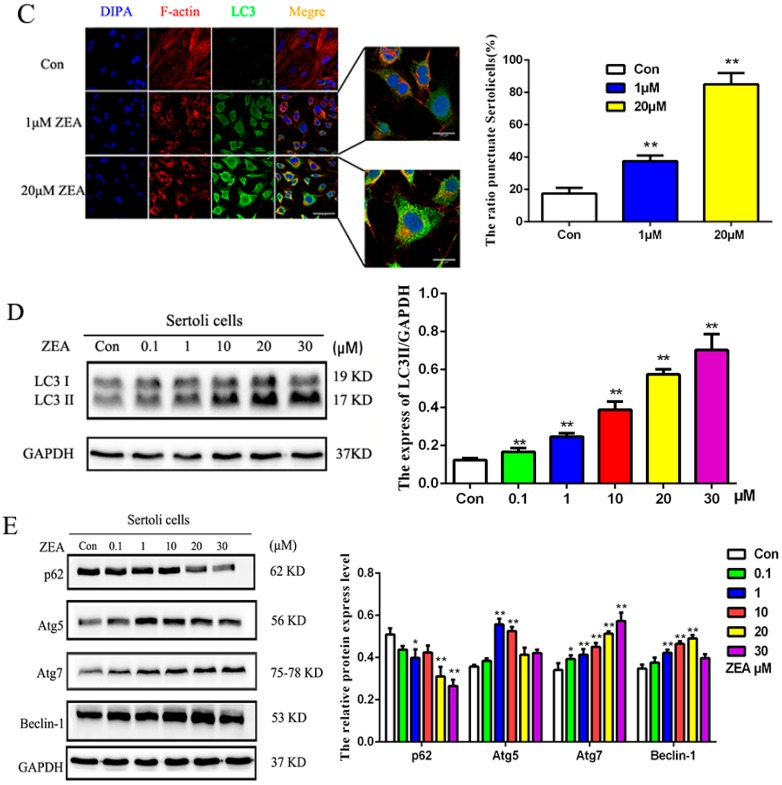
ZEA induced autophagy in SCs. (**A**) SCs treated without (**a**) or with 20 μM ZEA 6600× ((**b**) Low power; (**c**) High power). Autophagic vacuoles are indicated by white arrows; (**B**) Autophagy observed by fluorescence microscopy in which monodansylcadaverine-labeled bubble vesicles in the cytoplasm or perinuclear region are clear and visibly dotted (100×); (**C**) SCs in the logarithmic phase incubated at a concentration of 0 (control group), 1, or 20 μM ZEA for 24 h and then incubated with LC3 antibody using the observed technique (64×); (**D**,**E**) SCs treated with indicated concentrations of ZEA for 24 h and subjected to immunoblot analysis for the detection of LC3, Beclin1, p62, and autophagy-related genes Atg5 and Atg7 levels. * *p* < 0.05, ** *p* < 0.01 versus control.

To further confirm whether cytoplasmic vacuoles seen by inverted-phase contrast microscopy are related to autophagy, we divided the SCs into the ZEA treatment group (20 μM) and the control group for 24 h. No obvious distribution of autophagic vacuoles or cytoplasm was observed under electron microscopy in the control group (Figure 3A-a). While for the 20 μM ZEA treatment group, under the same situation, typical autophagic vesicles and bilayer membrane structures were visible (Figure 3A-c), showing autophagy lysosome (Figure 3A-b,A-c). These results further demonstrate that ZEA can induce morphological autophagy in SCs.

MDC is a fluorescent dye that can be absorbed by cells and displayed on autophagy vesicles. Autophagy was observed by fluorescence microscopy MDC-labeled bubble vesicles in the cytoplasm or perinuclear region dotted with a clear structure. Thus, MDC can be used to evaluate the level of autophagy in cells according to changes in the granular cells under fluorescence microscopy. As the output in Figure 3B, compared with the control group, as ZEA concentration increased, fluorescence density and the number of MDC-labeled autophagic particles increased as well. As it turns out, the ZEA could induce the formation of autophagy in a dose-dependent manner within a certain concentration range.

At the same time, to detect the effect of ZEA on SCs in the LC3 accumulation point, cells in each group were incubated with LC3 antibody (Figure 3C). Under fluorescence microscopy, the control group didn’t display any significant accumulation, while the ZEA treatment groups at concentrations > 10 μM showed LC3 fluorescence signaling and significantly increased numbers of fluorescent cells (** *p* < 0.01). The fluorescence intensity of individual cells in a certain concentration range increased in a dose-dependent manner. These results indicated that ZEA treatment could increase autophagy in SCs to some extent.

LC3 expression was detected by Western blotting (Figure 3D). The Western blot results showed that LC3 protein expression increased as ZEA concentrations increased in vitro (** *p* < 0.01; * *p* < 0.05) in a dose-dependent manner. The consistent detection and immunofluorescence results of the LC3 technique indicate that ZEA treatment can promote autophagy in SCs.

After treatment with different concentrations of ZEA treatment, ubiquitin binding protein p62 expression was downregulated (* *p* < 0.01; Figure 3E), However, the changes in Beclin-1, Atg5, and Atg7 protein expression were similar with that of LC3 II protein expression (** *p* < 0.01; * *p* < 0.05). Our results further contend that ZEA treatment could increase autophagy in SCs.

### 2.4. ZEA Induced Autophagy through Inhibiting the PI3K/Akt/m TOR Signaling Pathway in SCs

**Figure 4:**
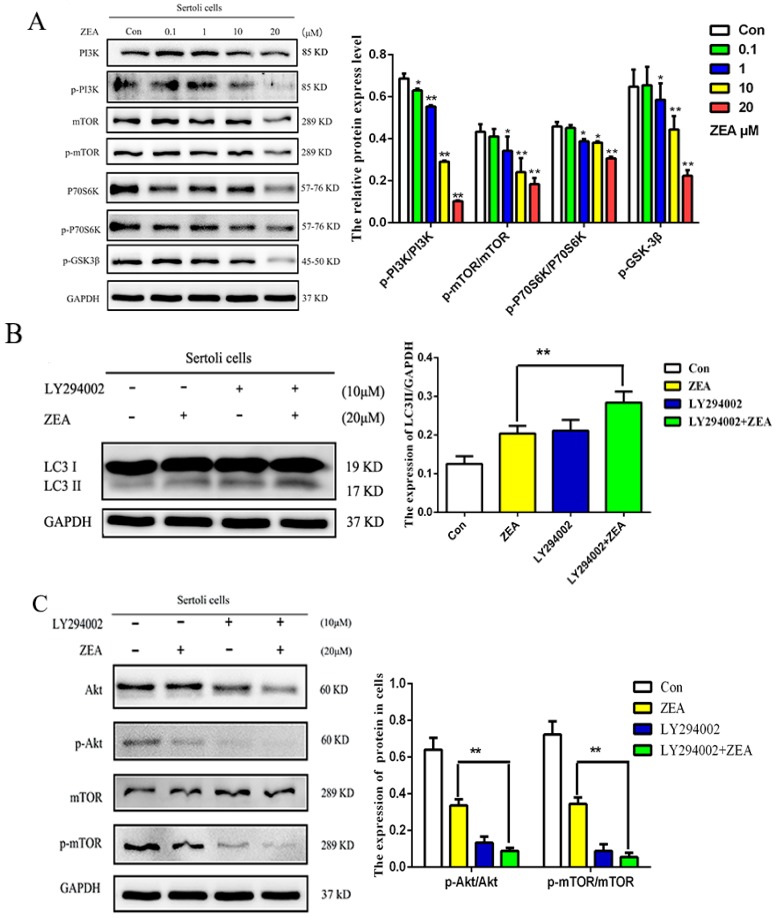

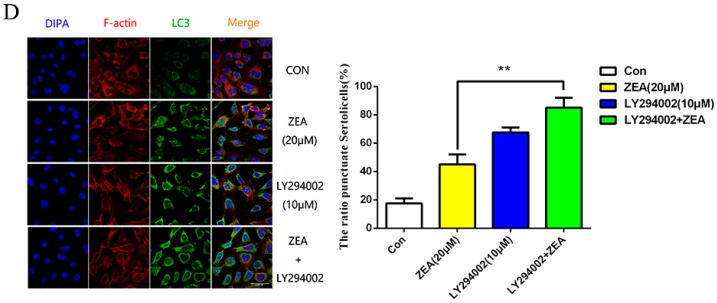
The role of the PI3K/Akt/m TOR signaling pathway in autophagy induced by ZEA. (**A**) The cells in the logarithmic phase were treated with different concentrations of ZEA for 24 h, Western-Blot detected the expression of key proteins phosphorylation levels in the PI3K/Akt/m TOR signaling pathway (* *p* < 0.05, ** *p* < 0.01); (**B**,**C**) Expression levels of p-AKT/AKT, p m TOR/m TOR, and LC3II/LC3I after adding PI3K inhibitors (LY294002) in the experiments (** *p* < 0.01); (**D**) Effects of PI3K inhibitor on point phenomenon of SCs treated with the ZEA.

The SCs in the logarithmic phase were disposed with different doses of ZEA for 24 h, Western-Blot detected the expression of key proteins phosphorylation levels in the PI3K/Akt/m TOR signaling pathway (PI3K, m TOR, and P70S6K). As shown in Figure 4A, within a certain concentration range, with the increase of ZEA concentration, the phosphorylation levels of PI3K, m TOR, and P70S6K decreased gradually. Compared with the blank group, when the concentration of ZEA was higher than 1 μM, the phosphorylation level of PI3K and P70S6K were significantly decreased (** *p* < 0.01), and the phosphorylation level of m TOR protein was significantly reduced (* *p* < 0.05), these results indicate that ZEA can induce autophagy by activating PI3K/Akt/m TOR signaling pathway.

To further clarify the role of the PI3K/Akt/m TOR signaling pathway in autophagy, after the PI3K inhibitor (LY294002) was pretreated, it was observed under the fluorescence microscope, as shown in Figure 4D, Compared with the ZEA treatment group, the fluorescence signal intensity of LC3 in LY294002 and ZEA was significantly increased (* *p* < 0.05); the increase of fluorescence signal cells increased gradually (* *p* < 0.05), and the fluorescence accumulation was more obvious. The Western blot results showed in Figure 4B,C, compared with the ZEA-only treatment group, the LY294002 and ZEA combination treatment caused markedly decreased expression levels of p-AKT/AKT and p-m TOR/m TOR (** *p* < 0.01), while the expression of LC3II/LC3I was significantly increased (** *p* < 0.01). These results suggested that PI3K/Akt/mTOR signaling pathway exerted a negative regulatory role in autophagy which ZEA induced in SCs.

### 2.5. The Role of Autophagy in ZEA-Induced G2/M Arrest in SCs

**Figure 5:**
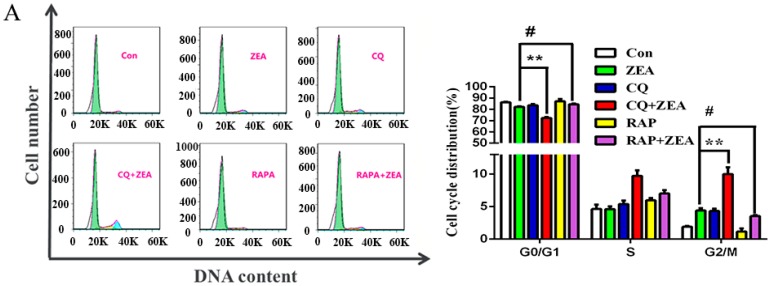

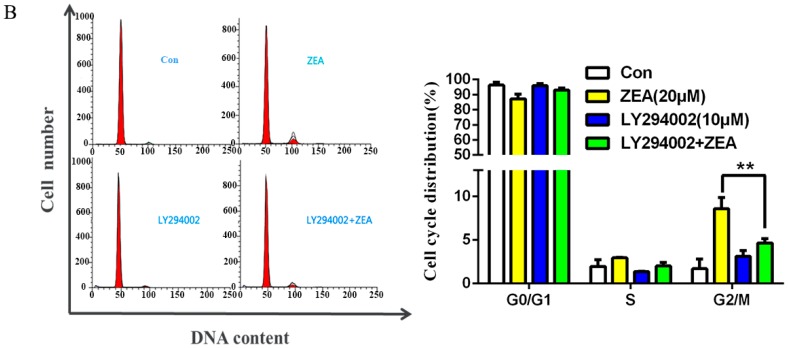
Influence of autophagy in ZEA-induced G2 arrest, a process in which autophagy plays a protective role (**A**,**B**). (**A**) When autophagy inhibitor (CQ) and autophagy promotor (RAPA) were added to the cells followed by treatment with 10 μM ZEA for 24 h, the cell cycle changes were detected by flow cytometry; (**B**) Effects of the PI3K/Akt/m TOR signaling pathway on ZEA induced the distribution of cycle in SC cell (* *p* < 0.05,** *p* < 0.01).

When the cells were disposed with 10 μM ZEA for 24 h and autophagy inhibitor (CQ) and autophagy promotor (RAPA) were then added, cell cycle changes were detectable by flow cytometry (Figure 5A). Compared with the ZEA-only group, after using autophagy inhibitor CQ, the percentage of SC in the G2/M phase was increased significantly (* *p* < 0.05). After co-treatment with RAPA, the proportion of SCs in the G2/M phase was decreased significantly (* *p* < 0.05). These results have indicated that autophagy could reverse the G2/M phase arrest of SCs cell cycle induced by ZEA.

To further clarify the effect of PI3K/Akt/m TOR pathway in ZEA-induced G2 arrest of SCs, the cells in the logarithmic phase were pre-treated with PI3K inhibitor (LY294002) for 30 min and then exposed to ZEA for another 24 h, the cell cycle changes were detected by flow cytometry. The results are shown in Figure 5B, Compared with the ZEA-only group, after the addition of PI3K inhibitor (LY294002), the percentage of SCs in the G2/M phase decreased significantly (* *p* < 0.05). These results have shown that using the PI3K inhibitor (LY294002) to promote the level of autophagy could also reverse the cell cycle arrest induced by ZEA in SCs.

### 2.6. The Role of Autophagy in the Expressions of Cell Cycle Regulatory Protein in SCs

**Figure 6:**
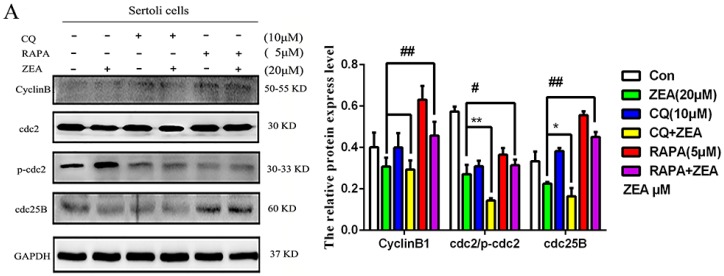

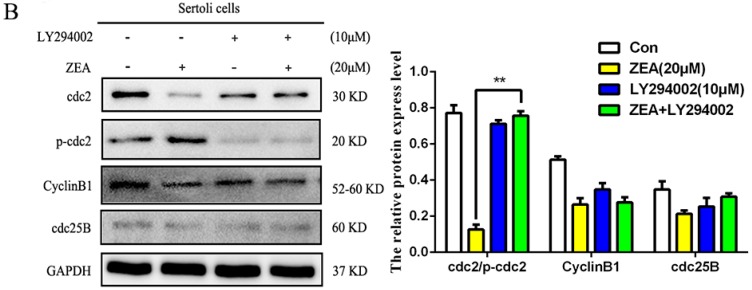
Influence of the autophagy and PI3K/Akt/m TOR signaling pathway in ZEA-induced cell cycle regulatory protein expression of SCs. The activation of the PI3K/Akt/m TOR signaling pathway could partly reverse the ZEA-induced arrest of SC in the G2/M phase, which through modulating the activity of cyclin kinase cdc2 (**A**,**B**). (**A**) Effects of autophagy on the protein expression of SCs in the G2/M phase; (**B**) Effects of PI3K inhibitors on the expression of G2/M phase symbolic proteins (*/# *p* < 0.05, **/## *p* < 0.01 versus the treatment group).

In order to detect the function of autophagy in the expressions of the G2/M transition associated proteins including cdc2 and Cyclin B1 in SCs, the autophagy inhibitor and promotor CQ and RAPA, and PI3K inhibitor (LY294002) were used in this study. As shown in Figure 4B, compared with the ZEA-only treatment group, the CQ and ZEA combination treatment resulted in markedly decreased expressions of the G2/M transition associated proteins including cdc2, cdc25B, and Cyclin B1. In addition, the ZEA-induced increased expression levels of cdc2/p-cdc2, cdc25B, and cyclin B1 were restored by RAPA pretreatment (Figure 6A). Compared with the ZEA-only treatment group, the LY294002 and ZEA combination treatment resulted in markedly enhanced activity of the G2/M phase critical kinase cdc2 (* *p* < 0.05). The expressions of CyclinB1 and cdc25B were different, but the difference was not significant (Figure 6B). These results have indicated that the inhibition of the PI3K/Akt/m TOR signaling pathway could partly reverse the ZEA-induced arrest of SCs in the G2/M phase, which through modulating the activity of cdc2.

## 3. Discussion

In this study, we employed rat primary SCs to investigate the effects and the molecular mechanisms of autophagy and PI3K/Akt/m TOR signaling pathway on male reproduction in vitro. We found that ZEA significantly inhibited SCs proliferation at certain concentrations and led to cell cycle arrest in the G2/M phase. The regulation of cell cycle is a multi-level and complex multi-step process [25]. Several researches have revealed that the early toxic effects of mycotoxins such as fusarium B1, ZEA, and deoxynivalenol (DON) are caused by apoptosis and proliferation inhibition or cell cycle arrest in high concentration, induce a death of germ cells [26,27].

Of note, cell cycle regulation is a very complex process that involves multiple signaling pathways and cytokines [28]. The key mitosis-promoting factor which regulated the transition from G2 to M phase is in part by Cyclin B1 and cdc2. Thereinto, the phosphorylation state of cdc2 inhibited the activity of Cyclin B1/cdc2 complex, an important enzyme that promotes conversion to the G2/M phase; when cdc25B is activated, it can cause cdc2 dephosphorylation, which enhanced the activity of Cyclin B1/cdc2 and stimulated the G2 conversion. Therefore, when G2/M arrest occurs, due to cdc25B activity inhibition, the higher-level phosphorylation state of cdc2 maintained and the activity of Cyclin B1/cdc2 complex is inhibited [29,30]. Moreover, p21 is a downstream mediator of the tumor suppressor p53 in stress conditions, which also negatively regulated the complex activation of Cyclin B1/cdc2 [31,32]. The results of our research showed that ZEA increased the protein expression levels of p53 and p21 (Figure 2B), and the expressions of cell cycle related proteins decreased significantly (Figure 2B). The expression of p-Histone H3 was decreased significantly in the mitotic phase (Figure 2C). These results suggest that ZEA inhibits SCs proliferation and is closely related to G2/M arrest.

It has been known for many decades that autophagy, a conserved lysosomal degradation pathway, is highly active during cell differentiation and development, and it regulates the intracellular homeostatic mechanism that mediates protein and organelle degradation. Studies [33,34,35] have shown that autophagy regulates the intracellular homeostatic mechanism that mediates protein and organelle degradation. Autophagy can be induced through multiple interconnected pathways, such as the MAPK, PI3K-AKT-mTOR, and ATP-AMPK-m TOR signaling pathways [36,37]. In our study, the immunofluorescence analyses show that as ZEA concentration increased, the LC3 accumulation in SCs significantly increased and the number of acidic vesicles increased (Figure 3C). Thus, these results demonstrated that ZEA can trigger autophagy in SCs. To further clarify the role of the PI3K/Akt/m TOR signal pathway in autophagy, we use LY294002 Inhibitor treatment. Results indicate that PI3K/Akt/m TOR signaling pathway plays a negative regulatory role in autophagy which ZEA induced in SCs.

Autophagy stabilizes the intracellular environment and maintains cell survival through balancing anabolism and catabolism [38]. The PI3K-Akt-mTOR signaling pathway is also a major signal transduction cascade involved in cellular metabolism, proliferation, and survival, and plays an important role in autophagy [39]. Thereinto, autophagy-related proteins such as Akt regulate many physiological processes within cells, promote cell division and proliferation, and play an important role in the cell cycle [40]. m TOR is one of the important regulatory factors in autophagy, which can sense the nutritional status and cell stress [41]. We supposed that there exists a forceful and intricate relationship between cell cycle and autophagy under different circumstances, to further investigate the effect of autophagy and PI3K/Akt/m TOR pathway on ZEA-induced G2 arrest of SCs, we add chloroquine (CQ), PI3K specific inhibitor (LY294002) treatment, and rapamycin (RAP) promoter treatment. The results are shown in Figure 5A,B, compared with the ZEA-only treatment group, we found that the percentage of SCs in the G2/M phase decreased significantly after the addition of PI3K inhibitor (LY294002). Western blot results showed that (Figure 6A,B) the expressions of Cyclin B1, ccd2, and cdc25B increased significantly when promoting autophagy, though compared with the ZEA treatment group, the LY294002 and ZEA combination treatment resulted in markedly enhanced cdc2 activity of the G2/M phase, the expressions of CyclinB1 and cdc25B were different, but the difference was not significant. These results indicate that the defects of autophagy could lead to cell cycle arrest in the G2/M phase; the inhibition of the PI3K/Akt/m TOR signal pathway could partly reverse the ZEA-induced arrest of SCs in the G2/M phase, through modulating the activity of cdc2.

## 4. Conclusions

**Figure 7:**
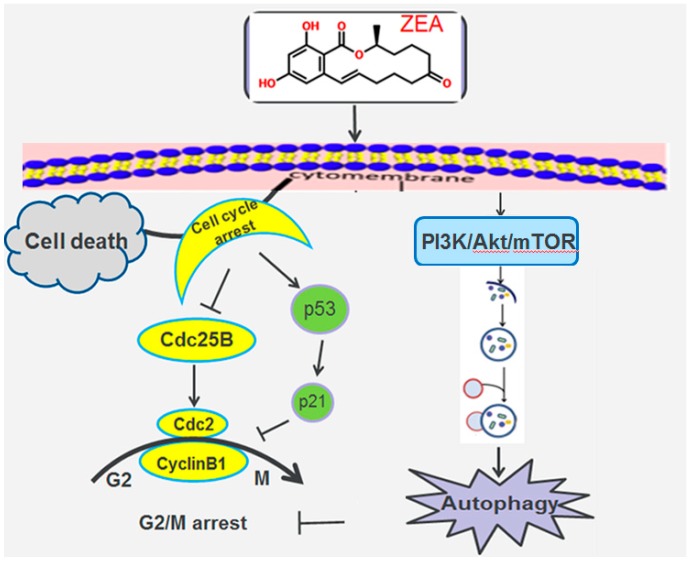
Schematic showing the protective role of autophagy in response to the ZEA–induced G2/M cell cycle arrest of SCs.

In summary, this study suggests that ZEA could inhibit the proliferation through arresting the cell cycle arrest in the G2/M phase. ZEA could induce autophagy through inhibiting the PI3K/AKT/m TOR signaling pathway in SCs. Inhibiting PI3K/Akt/m TOR signaling pathway can partially reverse the ZEA-induced arrest of SCs in the G2/M phase. Thus, to some extent, the ZEA-induced autophagy plays a protective role in this process (Figure 7).

## 5. Materials and Methods

### 5.1. Reagents

Zearalenone (ZEA), chloroquine (CQ), rapamycin (RAP), and dansylcadaverine (MDC) were purchased from Sigma-Aldrich (St. Louis, MO, USA); PI3K specific inhibitor (LY294002) were obtained from MCE (Shanghai, China); and the cell cycle assay propidium iodide (PI) were purchased from Becton Dickinson Company (BD, Franklin Lakes, NJ, USA).

The polyclonal antibodies against Beclin-1 (Ref. No.: 07/2016), LC3 (Sigma, L7543), Atg5 (Ref. No.: 09/2015), GAPDH (Ref. No.: 12/2016), HRP- conjugated goat anti-rabbit IgG (Ref. No.: 10/2016) were purchased from Cell Signaling Technology (Danvers, MA, USA); Enhanced chemiluminescence (ECL) solution was obtained from Thermo Fisher Scientific (Waltham, MA, USA). All the other chemicals and reagents were analytical grade and were obtained commercially.

### 5.2. Cell Cultures

All experimental procedures were conducted in accordance with the recommendations in the Guide for the Care and Use of Laboratory Animals of the National Research Council and were approved by the Animal Care and Use Committee of Yangzhou University (Yangzhou University Medical Center, Approval ID: SYXK (Su) 2017-0044). 18–21 days male Wistar rats were provided by Yangzhou University Medical Center Rats primary SCs were cultured at 37 °C in 5% CO_2_ in Dulbecco’s Modified Eagle Medium/F12 supplemented with 10% fetal bovine serum and antibiotics (100 U/mL penicillin G and 100 µg/mL streptomycin). ZEA was dissolved in dimethylsulfoxide (DMSO) and stored at −20 °C.

### 5.3. Cell Proliferation Assay

Cell proliferation was analyzed using a Cell Counting Kit-8 (CCK-8) (Dojindo, Japan). Cells were plated at a density of 1 × 104 per well in a 96-well plate, after exposure to ZEA at different concentrations (0, 0.1, 1, 10, 20, 40, 60, and 80 μM) for 24 h. CCK-8 solution (10 μΜ) was added to each well, the culture plate was placed in an incubator for 2–4 h, the plate was examined by an enzyme-linked immunosorbent assay tester, and absorbance was determined at 450 nm.

### 5.4. Cell Cycle Assay

To verify the effects on cell cycle distribution by ZEA and the percentage of cell cycle, the cells were examined by using the cell cycle detection kit. Before cell cycle determination, the SC were cultured with various concentrations of ZEA (0, 0.1, 1, 10, 20, 30 μM) for 24 h, and then suspended in 75% ethanol and fixed overnight at 4 °C. Then the cells were treated with 25 μg/mL propidium iodide (PI) for 15 min at room temperature in the dark. The proportions of cells in the G0/G1, S, and G2/M phases were determined by examining the intensity of PI fluorescence with flow cytometer using an argon laser and 570-nm bandpass filters.

### 5.5. Western Blotting Analysis

SCs in the logarithmic phase were cultured in a 100-mm dish, after exposure to different treatment group for 24 h. Proteins were extracted with cell lysis buffer on ice, then the lysis buffer was collected and centrifuged, each sample was diluted to the same protein concentration. Equal amounts of protein (40 μg/lane) was separated on 6–15% SDS–PAGE and then transferred to PVDF membranes (Millipore Corporation, Bedford, MA, USA). After incubated in blocking buffer for 2 h at room temperature. The membranes were probed with the indicated primary antibodies at 4 °C overnight, washed and then incubated with goat anti-rabbit/ mouse secondary antibodies for 2 h at room temperature. After extensive washed with Tris-buffered saline + Tween 20, they were visualized and analyzed with an ECL detection system.

### 5.6. Transmission Electron Microscopy (TEM)

To observe the cell ultrastructure changes and autophagy formation by transmission electron microscopy, the cells were inoculated into the dish and treated with 0 or 20 μM ZEA for 24 h. All suspended and adherent cells were collected. Using 0.25% trypsin, the cells were digested into a single cell suspension and centrifuged at 1000 r/min for 10 min. After precooling, the supernatant was washed twice with cold phosphate buffered saline (PBS), 2.5% glutaraldehyde was slowly added to prevent dissolution of the cells, and the suspension was fixed at 4 °C for 90 min. The cells were washed again with PBS, fixed with 1% osmium tetroxide at 4 °C for 30 min, and rinsed again with PBS. Finally, the cells were dehydrated with a series of ethanol (50–100%) (10%) and pure acetone and then embedded into EPON 812 resin. The embedded block was cut into thin sections, stained with uranyl acetate and lead citrate Zi, and observed under electron microscopy.

### 5.7. Immunofluorescence Microscopy

The SC were inoculated into the pre-placed six-well slide plates at 37 °C and 5% CO_2_ incubator training overnight. After exposure to different treatment group for 24 h, and fixed with 4% paraformaldehyde at 4 °C for 30 min, and after three washes with PBS, the cells were incubated with 5% BSA sealing solution at room temperature for 30 min. Then cells were incubated with LC3 primary antibody overnight at 4 °C, After washing, the cells were labeled with goat anti-rabbit IgG secondary antibody conjugated with FITC for 1 h, After washing again, nucleus was stained with DAPI for 15 min. Images were obtained by confocal microscope.

### 5.8. Statistical Analysis

The results are presented as the mean ± standard deviation (SD). Statistical data comparisons among groups were performed using a non-parametric, SPSS one-way analysis of variance, with *p* < 0.05 considered statistically significant. Each experiment was performed at least in triplicate.
